# The Effect of Mindfulness-Based Interventions on Tinnitus Distress. A Systematic Review

**DOI:** 10.3389/fneur.2019.01135

**Published:** 2019-11-01

**Authors:** Maaike M. Rademaker, Inge Stegeman, Krysten E. Ho-Kang-You, Robert J. Stokroos, A. L. Smit

**Affiliations:** ^1^Department of Otorhinolaryngology–Head and Neck Surgery, University Medical Center Utrecht, Utrecht, Netherlands; ^2^University Medical Center Utrecht Brain Center, University Medical Center Utrecht, Utrecht, Netherlands

**Keywords:** tinnitus, mindfulness, cognitive behavioral therapy, depression, anxiety, MBCT, MBSR

## Abstract

**Objectives:** With this systematic review we aim to provide an overview of the evidence of the effect of Mindfulness Based Interventions (MBIs) on (1) tinnitus distress and (2) anxiety and/or depression in tinnitus patients.

**Methods:** We conducted a systematic search in PubMed Medline, EMBASE and PsycInfo combining the terms and synonyms of “Tinnitus” and “Mindfulness.” The most recent search was performed on December 4th 2018. We wrote this systematic review according to the Preferred Reporting Items for Systematic Reviews and Meta-Analyses (PRISMA). Two independent authors identified studies, assessed the risk of bias and extracted data. Studies were considered eligible if they included adults with tinnitus, performed a protocolled MBI and measured tinnitus distress with validated questionnaires. Studies were appraised with either the Cochrane Risk of Bias tool or the MINORS criteria, depending on their design.

**Results:** The systematic search yielded seven articles (425 patients). Three randomized controlled trials (RCTs), three cohort studies and one comparative controlled trial. Different types of MBIs, including MBCT and MBSR, were assessed with various questionnaires. Two of three RCTs showed a statistically significant decrease in tinnitus distress scores directly after treatment in the mindfulness group compared to the control group. Six of seven studies showed statistically significant decrease in tinnitus distress scores directly after mindfulness therapy. One of three RCTs showed a statistically significant improvement of depression questionnaire scores after MBI compared to the control group directly post treatment.

**Conclusions:** A decrease of tinnitus distress scores in MBIs can be observed directly post-therapy based on moderate to high quality studies. This was found regardless of the heterogeneity of patients, study design, type of MBI and outcome assessment. Two out of three RCTs found clinically relevant decreases in tinnitus distress scores. No effect of MBIs was observed for depression and anxiety in tinnitus patients. Long term effects remain uncertain. Mindfulness may have a place in tinnitus therapy, although the long term effects need to be studied.

## Introduction

Tinnitus is the awareness of a ringing, buzzing or hissing sound in the ears or head without an external stimulus (1). The reported prevalence ranges between 10 and 15% (1). However, the experienced hindrance of tinnitus differs among patients: whilst some consider it negligible and grow accustomed to it, others are distressed and cope with a high level of disease. In 5% of the population mild to moderate disturbance by tinnitus is reported, while quality of life is severely reduced in 1–2% of tinnitus sufferers (1–4).

Tinnitus distress is described as a “multidimensional phenomenon that can be associated with problems such as difficulties with concentration, insomnia, or negative thinking which can amplify it in vicious cycles” (5)^p1^. Furthermore, suffering patients report hopelessness, exhaustion, feeling overwhelmed and even suicidal thoughts (2). Considering the broad range of difficulties patients with tinnitus experience and the diversity in associated co-morbidities, tinnitus perceptions (e.g., loudness, location), causal risk factors and levels of distress, tinnitus is considered a heterogeneous condition (6).

### Rationale

No curative treatment for tinnitus has been found thus far, and evidence for therapy that diminishes tinnitus distress is limited (7). Cognitive Behavioral Therapy(CBT) has been proven effective in improving quality of life in tinnitus patients (8). CBT is a structured, time limited psychological therapy which is based on the idea that a person's perception is influenced by personal experiences (8). It encourages patients to use cognitive and behavioral tasks to modify their response to thoughts and situations (9). There is great variation within the CBT approaches for tinnitus, even though common elements are found. One of these approaches is Relaxation Therapy (RT), based on the work by Lars-Göran Ost. It teaches patients to relax rapidly, in order to reduce anxiety (10). In recent years the so-called third wave of CBT has gained more interest. Third-wave methods focus more on the patient's relationship to thoughts and emotions than on thought content, emphasizing acceptance and mindfulness (11).

Mindfulness can be defined as: “the effort to intentionally pay attention, non-judgementally, to present-moment experience and sustain this attention over time” (12)^p123^. It is the practice of intentionally and openly attending to what is happening in the present; to not lose focus because of thoughts or sensations, nor to judge them (13, 14). Mindfulness was introduced to modern western medicine in 1979 by Jon Kabat-Zinn (15). He adapted and adopted traditional ‘eastern' meditative techniques into a program systematically training mindfulness, known as Mindfulness Based Stress Reduction (MBSR) (15, 16). Since the introduction of MBSR, different types of courses that integrate mindfulness techniques in contemporary psychological practice have been developed. These are collectively referred to as Mindfulness-Based Interventions (MBIs) (17). MBIs emphasize formal meditation and aim to help incorporate mindfulness in daily life (17). It involves control of attention and acceptance of present moment experiences (14). Segal et al. adapted the MBSR program to combine CBT and mindfulness techniques in order to treat relapsing depression; this was called Mindfulness Based Cognitive Therapy (MBCT) (18, 19). Both interventions (MBSR and MBCT) consist of 8 weekly group classes that last 2–2.5 h with a potential, one-time, all-day retreat. Homework and exercises to integrate mindfulness technique in normal daily life play an important role in both interventions (17). MBIs have been proven effective in treating several conditions such as stress, anxiety, depression, and chronic pain, all symptoms that are also associated with tinnitus (20–23).

Mindfulness is thought to work by changing the perception of negative experiences, making them less emotionally destructive. Patients are believed to become more aware and less preoccupied by physical or mental sensations after a MBI (14, 24). Avoidance is associated with tinnitus severity and distress. Acceptance, a key element of mindfulness, is the exact opposite of avoidance, which could help tinnitus sufferers to benefit from a MBI (25, 26).

### Objectives and Research Question

The primary objective of this study is to systematically assess the effect of MBIs on tinnitus distress in the literature. Our secondary objective is to assess the effect of MBIs on depression and/or anxiety in tinnitus patients.

## Methods

### Study Design

We considered randomized controlled trials (RCTs), and observational studies [retrospective and prospective cohort studies and case series (*n* ≥ 5)]. We included studies if they included adults with tinnitus, with all types of protocolled MBIs.

The primary outcome measure was tinnitus distress measured with a validated questionnaire. The secondary outcome measures were depression and/or anxiety measured with a validated questionnaire.

### Participants, Interventions, Comparators

We searched for studies in accordance with the PICO search method (Patients, Intervention, Comparison, and Outcome). P: adults with tinnitus, I: protocolled MBI, C: any other therapy or no therapy and O: tinnitus distress, depression and/or anxiety measured with validated questionnaires. Only articles with original data were included for full text screening.

### Systematic Review Protocol

We did not create a review protocol. This review was written according to the Preferred Reporting Items for Systematic Reviews and Meta-Analyses (PRISMA) (27).

### Search Strategy

We conducted a systematic search on December 4th, 2018. The search was created and conducted by the review team (MR, KH, DS, IS). There were no restrictions regarding publication year, language, or publication status.

Electronic database searches were performed in PubMed, EMBASE, and PsycInfo. The search terms can be found in Supplementary Material. Search terms were based on the PICO search method, in which a combination of synonyms for the *P* (patients) and the *I* (interventions) were searched. Reference lists and citations of included articles were scanned to identify possible relevant studies. ClinicalTrials.org was searched for ongoing trials and protocols.

### Data Sources, Studies Sections, and Data Extraction

Two authors (MR and KH) selected applicable studies. Title-abstract and full-text screening were performed by two independent authors (MR and KH) and based on predefined inclusion and exclusion criteria (Table 1).

**Table 1:** Inclusion and exclusion criteria.

**Inclusion criteria**	**Exclusion criteria**
Adults with tinnitus	Treatment with Acceptance and Commitment therapy
Protocolled MBI	Case reports (*n* ≤ 5)
Tinnitus distress	Systematic review, meta-analysis
	Letter to the editor
	Animal studies
	Conference proceedings

Original data from included articles were extracted by two authors (MR and IS). The following data was extracted, if provided: (1) total number of participants in each study, (2) total number of patients in each group, (3) intervention type and frequency, (4) outcome data pre-intervention, post-intervention and at 3 and 6 months follow up, (5) mean difference and *p*-values. If data was not provided, it was extracted from figures if possible. If mean differences were not provided, they were calculated if possible.

Differences on the questionnaires were considered clinically relevant if they reached a published minimal clinical important difference (MCID) [German Tinnitus Questionnaire (TQ) 12 points, Tinnitus Handicap Inventory (THI) 7 points, Tinnitus Functional Index (TFI) 13 points] (28–30).

Selected studies were critically appraised by two independent authors (MR and KH). Disagreements were solved by discussion. We included RCTs and observational studies. The RCTs were assessed with the Cochrane Risk of Bias Tool (31). The observational and the comparative controlled trial were assessed with the MINORS tool. Items were scored as 0 (not reported), 1 (reported but inadequate) or 2 (reported and adequate) (32).

### Data Analysis

Meta-analyses were performed in case of homogenous outcomes.

## Result

### Study Selection and Characteristics

The electronic search of PubMed, EMBASE, and PsycInfo yielded 52 results. After removal of 18 duplicates, 34 studies were screened on title and abstract. Title/abstract screening resulted in 10 articles. Three articles were excluded: one was a duplicate (33), one was a letter to the editor (34), and one did not use a validated outcome measure in addition to including supplementary therapy to a MBI (35). We included 7 studies in this review (*n* = 425 patients, range of 8–182 patients per study) published between 2007 and 2017 (13, 24, 36–40) (Table 2).

**Table 2:** Flowchart.

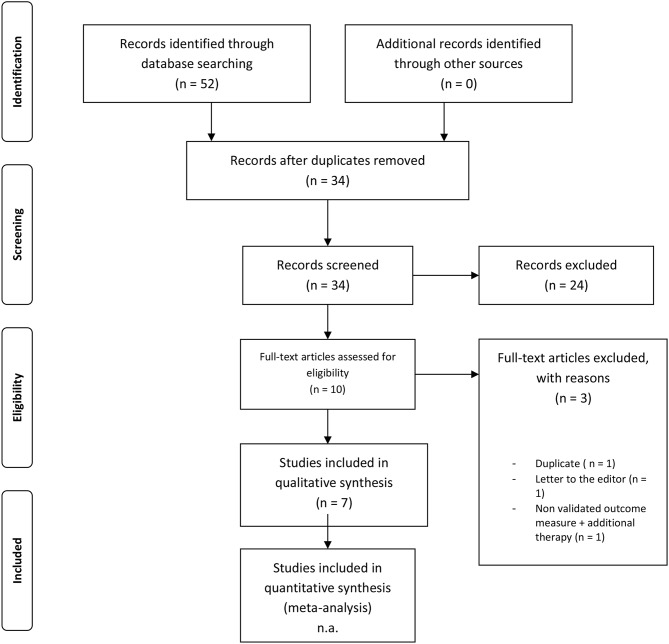

Three RCTs (35–37) one comparative controlled trial (13) and three cohort studies (24, 39, 40) were included in our review. Six out of the seven studies assessed the outcome only at the end of therapy, while one study had a follow up at 6 months (RCT) (36). Characteristics of the included studies are summarized in Table 3. Different types of questionnaires were used to measure outcomes of tinnitus distress, anxiety or depression; these are described in Table 4. In the article by Kreuzer et al. post therapy was considered to be the measurement after week 24 (38). Data on the TQ from Sadlier was deducted by manual measurements (13).

**Table 3:** Characteristics of included studies.

**References**	**Study design**	***N***	**Follow up**	**Minimum tinnitus duration**	**Mean duration of tinnitus at inclusion in months (± SD)**	**Intervention type**	**Schedule**	**Control type**	**Schedule**	**Outcomes**		
										**Tinnitus Distress**	**Anxiety**	**Depression**
McKenna et al. (36)	RCT	75	1, 6 M	>6 M	56 (± 104) (median, IQR)	MBCT	8 WKL × 120 min	RT^d^	8 WKL × 120 min	TQ	HADS-A	HADS-D
										TFI		
Arif et al. (37)	RCT	86		NR	62.4 (± 58.8)^a^ 78 (± 52.8)^b^	MBI	5 × 40 min in 15W	RT^d^	5 × 40 min in 15W	TRQ	HADS-A	HADS-D
Kreuzer et al. (38)	RCT	36		6 M	100.5 (± 119.1)^a^ 142.3 (± 116.2)^b^	MBI	2 × weekend & 4 × 120 min^e^	Waitlist	NA	TQ^G^	NR	BDI
										THI		
McKenna et al. (36)	Cohort	205	6 W	>3 M	NR	MBCT	8 WKL	NA	NA	TQ	NR	NR
Roland et al. (39)	Cohort	13	4 W	6 M	48 (NR) (median, IQR)^a^, ^c^	MBSR	8 WKL × 120 min & 1 × 180 min retreat	NA	NA	TFI	PROMIS-Anxiety	PHQ-9
										THI		
Gans (33)	Cohort	10		6 M	NR	MB (T) SR	8 WKL × 150 min	NA	NA	THI VAS-distress	HADS	HADS
											SCL-90-R Anxiety	SCL-90-R Depression
Sadlier (17)	CCT^*^	25		NR	102 (± 82.8)^a^ 96 (± 75.6)^b^	MBI	4 × 40 min^f^	Waitlist	NA	TQ	HADS-A	HADS-D

* *CCT, Comparative Controlled Trial*.

a *Intervention group*,

b *Control group*,

c *a median duration of bothersome tinnitus was reported of 1.8 years (0.6–10 years range)*,

d *RT, Relaxation Therapy*.

e *Group sessions at 2 training weekends, separated by an interval of 7 weeks (11 h/weekend) and in 4 further 2-h sessions (week 2, 9, 18, and 22)*.

f *Time range not specified. For abbreviation of outcome measures see Table 4. MBCT: mindfulness-based cognitive therapy, MBSR, mindfulness based stress reduction, MBI, Mindfulness Based Intervention; NR, not reported; NA, not applicable*.

G *German version, M, months; W, weeks; WKL, weekly; min, minutes*.

**Table 4:** Outcome measures: abbreviations, full names, range, and measure.

**Abbreviation**	**Full name (range)**	**Measures**
**Questionnaires to assess outcome**		
**TINNITUS DISTRESS**		
TFI	Tinnitus Functional Index (range 0–100)	Sleep (12%), auditory perception (12%), health (12%), impact on lifestyle (12%), psychological/emotional (36%), tinnitus specific effects (16%) (28)
THI	Tinnitus Handicap Inventory (range 0–100)	Sleep (4%), auditory perception (4%), health (4%), impact on lifestyle (20%), psychological/emotional (68%) (28)
TRQ	Tinnitus Reaction Questionnaire (range 0–104)	Sleep (4%), impact on lifestyle (19%), psychological (77%) (28)
TQ	Tinnitus Questionnaire (range 0–84) (29)	Sleep (10%), auditory perception (8%), health (13%), impact on lifestyle (12%), psychological/emotional (47%), tinnitus specific effects (10%) (28)
TQ—German translation	Tinnitus Questionnaire (range 0–84) (29)	NR
VAS	Visual Analog Scale (range 0–100)	NR
**ANXIETY**		
HADS-A	Hospital Anxiety and Depression Scale—Anxiety (range 0–21)	To detect state of anxiety (41)
PROMIS-anxiety	Patient Reported Outcomes Measurement Information System—Anxiety (range 36.3–82.7)	Severity of anxiety (42)
SCL-90-R-Anxiety	Symptom Checklist−90—Revised—Anxiety(range 10–50)	Extent of anxiety complaints (43)
**DEPRESSION**		
HADS-depression	Hospital Anxiety and Depression Scale—Depression (range 0–21)	To detect state of depression (41)
BDI	Becks Depression inventory (range 0–63)	Presence and severity of depressive complaints (44)
PHQ-9	Patient Health Questionnaire-9	Symptoms of depressive disorder (45)
SCL-90-R-Depression	Symptom Checklist−90—Revised—Depression (range 16–90)	Extent of depressive complaints (43)

Two studies used MBCT (24, 36) and two studies used MBSR, all according to the general standards for these treatments (as stated in the section Introduction) (39, 40). Gans et al. adapted the MBSR program to Mindfulness Based Tinnitus Stress Reduction (MBTSR), where the central focus was placed on psychoeducation related to co-occurring complaints in tinnitus patients. Class-time was focused on the awareness of sound and tinnitus perception (40).

Three other studies used different MBIs (13, 37, 38). Of these, Arif et al. administered “mindfulness meditation” conducted by a single experienced therapist, which consisted of five (40 min) sessions over a period of 15 weeks. The therapy included in-session exercises and homework (37). Kreuzer et al. administered a “mindfulness- and body-psychotherapy based group treatment”, conducted by a single experienced therapist. Participants received 2 weekends (11 h per weekend, 7 weeks apart) of therapy (including self-massage, breathing exercises, mindfulness meditation). Two weeks after each weekend and 11 and 15 weeks after the second training there was a 2 h review meeting. Homework was encouraged (38). Sadlier et al. administered a “mindfulness meditation cognitive behavioral therapy approach,” which was based on the work of Kabat-Zinn (15). Patients received four therapy sessions of 40 min each. The therapy involved a blend of approaches using CBT and mindfulness meditation. Mindfulness was introduced in the third session (13).

Relaxation Therapy (RT) was used as control group in two of the included RCTs; (10, 36, 37) McKenna et al. applied the RT in eight weekly sessions, while Arif et al. provided five sessions of RT in 15 weeks (36, 37). In the RCT by Kreuzer et al. a waiting list served as the control group, which was similar to the included comparative controlled trial by Sadlier et al. (13) and Kreuzer et al. (38).

### Risk of Bias Assessment

#### Cochrane Risk of Bias Assessment for Included RCTs

The items sequence generation, allocation concealment, incomplete outcome data, selective outcome reporting, and other sources of bias were scored as low risk of bias in all three RCTs (36–38). Blinding of participants and personnel scored high risk of bias in all three studies; this is inherent to the intervention. For outcome assessment McKenna et al. scored a low risk, the other two RCTs scored an unclear risk (Table 5).

**Table 5:** Cochrane risk of bias table.

**RoB item**	**Sequence generation**	**Allocation concealment**	**Blinding of participants and personnel**	**Blinding of outcome assessors**	**Incomplete outcome data**	**Selective outcome reporting**	**Other sources of bias**
**References**							
McKenna et al. (36)	–	–	+	–	–	–	–
Arif et al. (37)	–	–	+	?	–	–	–
Kreuzer et al. (38)	–	–	+	?	–	–	–

–*, Low risk of bias; +, High risk of bias; ?, Unclear risk of bias*.

#### MINORS Criteria for Included Non-randomized Studies

“Reported and adequate” was scored for the items a clearly stated aim, prospective collection of data and endpoint appropriate to the aim of the study in all four included non-randomized studies (13, 24, 39, 40). “Reported and adequate” was scored on adequate control group, contemporary groups and adequate statistical analyses for Sadlier, where other studies scored “not applicable” (13). The items unbiased assessment of the study endpoint and prospective calculation of the study size were all scored as “not reported” (Table 6).

**Table 6:** MINORS criteria.

**References**	**A clearly stated aim**	**Inclusion of consecutive patients**	**Prospective collection of data**	**Endpoints appropriate to the aim of the study**	**Unbiased assessment of the study endpoint**	**Follow-up period appropriate to the aim of the study**	**Loss to follow up less than 5%**	**Prospective calculation of the study size**	**Adequate control group**	**Contemporary groups**	**Baseline Equivalence**	**Adequate statistical analyses**	**Total**
Sadlier et al. (13)	2	1	2	2	0	2	1	0	2	2	1	2	17/24
McKenna et al. (36)	2	2	2	2	0	2	1	0	NA	NA	NA	NA	11/16
Roland et al. (39)	2	2	2	2	0	2	2	0	NA	NA	NA	NA	12/16
Gans et al. (40)	2	2	2	2	0	1	0	0	NA	NA	NA	NA	9/16

*0, not reported; 1, reported but inadequate; 2, reported and adequate*.

### Primary Outcome

Two out of three RCTs showed a significantly higher decrease in tinnitus distress after treatment in the mindfulness therapy group compared to the control groups (36, 37). Of these, the RCT by McKenna et al. compared MBCT to RT and found a statistically significant improvement on the TQ of (adjusted mean difference, AMD)−6.3 points (95% CI −11.5 to −1.2, *p* = 0.016) compared to RT, while no statistically significant difference was seen between both treatments by usage of the TFI (AMD−4.8 points (95% CI −12.2 to 2.5), *p* = 0.199] (36). The second RCT by Arif et al. compared a MBI to RT using the Tinnitus Reaction Questionnaire (TRQ) and found a statistically significant improvement of tinnitus distress after MBI compared to the control group (mean difference−8.2 point (95% CI −16.4 to −0.1, *p* = 0.047) (37). Of these studies only McKenna et al. included an outcome assessment at 6 months follow-up, where a superior effect of MBI was still seen over RT for the TQ score (AMD−7.2 points (95% CI −12.3 to −2.1, *p* = 0.006) and the TFI score (AMD−9.6 points (95% CI −17 to −2.3, *p* = 0.011) (36). The third RCT by Kreuzer et al., comparing MBI to a waiting list, did not find a statistically significant difference between both groups after treatment for the TQ and THI (38) (Table 7).

**Table 7:** Primary outcome measures.

**References**	**Questionnaire**	**Mindfulness- type**	**Pre**	**Post**	**FU**	**Control-type**	**Pre**	**Post**	**FU**
McKenna et al. (36)	TQ	MBCT	47.7 (13.8)	31.4 (16.1)^*^	28 (18.1)^*^ [6M]^a^	RT	48.1 (14.1)	**38.2 (14.3)**	35.6 (16.8) [6M]^a^
	TFI		60.6 (16)	**42.2 (19.2)**	37.2 (24.1)^*^ [6M]^a^		62.8 (15.8)	**49.2 (19)**	49 (21.1) [6M]^a^
Arif et al. (37)	TRQ	MBI	39.4 (15.4)	15.1 (13.1)^*^		RT	41.8 (17.7)	19.6 (13.2)	
Kreuzer et al. (38)	TQ^G^	MBI	34.6 (16.7)	**26.5 (16.3)**		WL	38.5 (14.7)	**33.1(16.6)**	
	THI		40.9 (21.7)	**27.3 (19.9)**			47.1 (17.5)	**41.3 (21.1)**	
McKenna et al. (36)	TQ	MBCT	42.5 (1.1)	**30.4 (1.21)**					
Roland et al. (39)	TFI	MBSR	39 (24-61)	**27 (6–60)**					
	THI		28 (20-50)	**28 (14–44)**					
Gans et al. (40)	THI	MB (T) SR	50.6 (15.2)	39 (21.8)					
	VAS-distress		59 (24.9)	36.9 (24.3)					
Sadlier et al. (13)	TQ	MBI	55 (NR)	**41 (NR)**		WL	54 (NR)		

*Bold numbers indicate statistical significance compared to the pre-therapy measurement*.

* *Indicates statistical significance of the mindfulness therapy compared to the control group. For Kreuzer (38) post therapy was considered to be the measurement after week 24. Please note, data from Sadlier on the TQ was deducted by manual measurements, data might not be accurate*.

G *German version*.

a *We were not able to deduct whether there was statistical significance within the randomization groups after 6 months compared to pre-therapy*.

Considering the effect of different mindfulness therapies, all seven randomized and non-randomized studies reported a lower score on the different tinnitus distress questionnaires post-treatment compared to pre-treatment scores. Six out of the seven studies demonstrated a statistically significant reduction after treatment with MBIs (Table 7).

### Secondary Outcome

Six out of the seven included studies reported outcomes of anxiety and depression measurement.

Only one out of three RCTs showed a statistically significant decreased depression score (Becks Depression Inventory, BDI) in the MBI group compared to the control group (waiting list) directly after treatment (*p* = 0.035) (38).

Considering the effect post-treatment vs. pre-treatment, all six studies showed lower absolute scores for anxiety and depression outcomes after a MBI on different questionnaires (Table 8). Only one study showed a statistically significant decreased depression score (BDI) after MBI [*p* = 0.024, calculated mean difference−3.8 (95% CI −7.3 to −0.3)] (38). Due to heterogeneous designs, interventions and outcome measures, we were not able to pool the data nor conduct a meta-analysis.

**Table 8:** Secondary outcome measures.

**Reference**	**Questionnaire**	**Mindfulness-type**	**Pre**	**Post**	**FU**	**Control-type**	**Pre**	**Post**	**Fu**
McKenna et al. (36)	HADS-A	MBCT	12.4(3.6)	9.2 (3.8)	9 (3.8) [6M]	RT	12.3 (4.1)	10.1 (3.9)	10.2 (3.7) [6M]
	HADS-D		8.4 (3.3)	6.2 (3.1)	5.6 (3.6) [6M]		9 (3.7)	7.5 (3.8)	7.5 (4.2) [6M]
Arif et al. (37)	HADS-A	MBI	7.0 (4.3)	4.6 (2.8)		RT	7.5 (3.5)	5.9 (4.0)	
	HADS-D		6.4 (4.4)	4.8 (3.0)			6.2 (3.0)	5.2 (3.8)	
Kreuzer et al. (38)	BDI	MBI	11.4 (8.4)	7.6 (5.7)^*^		WL	12.3 (6.9)	13.3 (8.7)	
McKenna et al. (36)		MBCT							
Roland et al. (39)	PROMIS-Anxiety	MBSR	NR	NR					
	PHQ-9		NR	NR^a^					
Gans et al. (40)	HADS	MB (T) SR	15.5 (6.5)	13.4 (7.2)					
	SCL-90-R Anxiety		63.4 (12.6)	54.9 (24.0)					
	SCL-90-R Depression		66.5 (12.3)	56.4 (24.4)					
Sadlier et al. (13)	HADS-A	MBI	9.4 (NR)^b^	7.9 (NR)^b^		WL	NR	NR	
	HADS-D		4.8 (NR)^b^	3.9 (NR)^b^			NR	NR	

*Bold numbers indicate statistical significance compared to the pre-therapy measurement*.

* *Indicates statistical significance of the mindfulness therapy compared to the control group. For Kreuzer (38) post therapy was considered to be the measurement after week 24*.

a *Roland et al. reported: “Although not clinically significant, the responses on the PHQ-9 assessed at the end of MBSR intervention changed from the baseline PHQ-9 scores with a median of 21 points and range from 3 points reduction to 1 point increase (P = 0.03). All patients had post-MBSR PHQ-9 scores of <15” (39)*.

b *Please note it was unclear whether the data presented is only the data from the direct-intervention group, or combined data from both groups*.

## Discussion

### Summary of Main Findings

In this study we assessed the effect of MBIs on tinnitus distress. Our search yielded seven articles, consisting of three RCTs, three cohort studies, and one comparative controlled trial. The three RCTs had a low risk of bias; the cohort studies and the comparative controlled trial had medium risk of bias.

Two out of three RCTs showed a statistically significant reduction in tinnitus distress directly after treatment in the mindfulness group compared to the control group (both RT).The observed changes in TFI scores in the RCTs by McKenna et al. and Arif et al. are considered clinically relevant by exceeding the MCID on the TFI (13 points) (28, 36, 37). Interestingly, the third RCT comparing the effect of a MBI to a waiting list group didn't show statistically significant differences (38). Besides differences in the comparison, we observed differences in timing and scheduling of MBIs. However, to what extent this can explain differences in outcome cannot be answered by our review.

In six out of seven included studies, a statically significant decrease of tinnitus distress scores was observed directly post treatment, regardless of MBIs, study designs and type of questionnaire (13, 24, 36–39). These decreases reached MCID in three out of six studies (36, 38, 40).

Long term outcome (6 months) was only assessed by one RCT (36). A persistent statistically significant decrease in tinnitus distress scores was observed after 6 months, compared to pre- and directly posttreatment scores (36). In an additional letter to the editor, Gans et al. reported a sustained effect of the MB (T) SR therapy 1 year after treatment in a small set of patients attending, subsequent follow-up assessment (34). This limited insight in the long term results of MBI on tinnitus distress hinders strong conclusions and needs further exploration.

There are several overlapping psychological, theoretical frameworks that attempt to explain the mechanisms of improved tolerance of tinnitus (9, 26, 46). The use of CBT, including third-wave approaches, is generally based on these models. These include habituation and cognitive techniques that intervene in dysfunctional responses (26). Tinnitus severity is associated with negative cognitions (47). Mindfulness is believed to increase cognitive and metacognitive awareness, whereby negative thoughts will become less emotionally destructive in tinnitus patients (9, 36). Additionally, the persistence of tinnitus can lead to conditioned fear responses such as avoidance, which is associated with tinnitus severity and distress (25, 26). By advocating acceptance rather than avoidance, mindfulness might be beneficial to tinnitus patients to lower tinnitus distress (26, 48).

In regard to the secondary outcome of anxiety and depression, one out of the three RCTs showed statistically significant decreased depression scores after MBI compared to the control group (waiting list) (38). Only one out of the six studies demonstrated a statistically significant reduction of scores on a depression questionnaire after MBI compared to the pre-treatment condition (38). Regardless of treatment group (mindfulness or control), the scores on the different anxiety/depression questionnaires were all relatively low at the pre-treatment measurement (reported range of pre-treatment HADS-A: 7.0–12.4, HADS-D: 4.8–8.4 (norm range 0–21, 8–10 = possible anxiety/depression)/pre-treatment BDI 11.4 (norm range 0–63, 0–13 = minimal depression) (Table 4).

In literature MBIs have been demonstrated to be effective in reducing depressive symptoms and anxiety for non-tinnitus patients (18). This can be beneficial for tinnitus patients as these symptoms are often related to the tinnitus complaints. No effects of MBIs were observed in our study, potentially due to the low anxiety and depression scores pre-treatment. A meta-analysis by Hofmann et al. showed that in patients with elevated levels of anxiety and depression but with other primary complaints, the effect size after a MBI is smaller than in patients with anxiety or depression as their primary complaint (21). This could also possibly explain the lack of effect of MBIs on anxiety and depression scores.

Some biases in the included studies need to be taken into consideration. Firstly, only one study investigated the effect of a MBI (MBCT) after 6 months. Even though the reduced tinnitus distress scores were maintained after 6 months, the effectiveness of MBIs over time remains uncertain (36). Secondly, there is a large heterogeneity in outcome measures used in the included studies, which hinders generalization of results. Even though all have been validated, each questionnaire addresses a slightly different domain of tinnitus and its impact (28). Nevertheless, the majority of included tinnitus distress questionnaires used in this review (TQ, TRQ, THI, and TFI) has a strong focus on the psychological/emotional domain. Thirdly, in this review the studied patient population is heterogeneous (e.g., minimum tinnitus duration at inclusion, mean tinnitus duration, different ranges of initial tinnitus distress at inclusion) with a large spread of outcomes after intervention. This also suggests a high heterogeneity in the different tinnitus patients and/or effects of therapy. Because one treatment will most likely not benefit all patients, a “subgroup” analysis might be needed to assess the therapeutical effects in relation to specific patient or disease related characteristics (6, 49). Fourthly, the heterogeneity in treatment protocols underlines the need for determining which elements are essential to successful MBIs for tinnitus.

## Conclusions

In this systematic review we found beneficial effects of MBIs on decrease in tinnitus distress scores, regardless of the heterogeneity of tinnitus patients, study designs, interventions and outcome measures. Two out of three RCTs found clinically relevant decreases in tinnitus distress scores. The included studies were of moderate to high quality. No effects of MBIs were observed for depression and/or anxiety. Conclusions concerning long term effects cannot be drawn, we therefore advise future trials to include a longer follow up.

## Author Contributions

MR, AS, KH, and IS designed the study, created and conducted the search. MR and KH selected the applicable studies, title-abstract, and full-text screening were performed by MR and KH. Original data from included articles were extracted by MR and IS. Risk of Bias was assessed by MR and KH. The manuscript was written by MR, KH, IS, RS, and AS critically revised the work and approved the final version to be published.

### Conflict of Interest

The authors declare that the research was conducted in the absence of any commercial or financial relationships that could be construed as a potential conflict of interest.
