# Supplementary material for: The Effect of Mindfulness-Based Interventions on Tinnitus Distress. A Systematic Review
Source: Front Neurol. 2019 Nov 1;10:1135. doi: 10.3389/fneur.2019.01135 (PMC6838968; doi:10.3389/fneur.2019.01135)
Supplement: Supplementary file 1 [file Table_1.DOCX]

Search strategy dd 04-12-2018

**Pubmed**

(((tinnitus[MeSH Terms]) OR tinnitus[tiab])) AND ((((Mindfulness[MeSH Terms]) OR mindfulness[tiab]) OR mbct[tiab] OR mbsr[tiab]))

**Embase**

(‘tinnitus’/exp OR ‘tinnitus’:ab,ti) AND (‘mindfulness’/exp OR ‘mindfulness’:ab,ti OR ‘mbct’:ab,ti OR ‘mbsr’:ab,ti)

**PsycInfo**

(mindfulness or MBSR or MBCT).ab,ti. AND Tinnitus.ab,ti.
